# Associated factors of sarcopenia among urban community-dwelling older adults in West Jakarta: A cross-sectional study

**DOI:** 10.51866/oa.594

**Published:** 2025-04-18

**Authors:** Yvonne Suzy Handajani, Yuda Turana, Kevin Kristian, Nelly Tina Widjaja, Aylenia Lysandra, Elisabeth Schröder Butterfill

**Affiliations:** 1 MPH, PhD, Ageing Research Centre, Atma Jaya Catholic University of Indonesia, Jalan Pluit Raya No.2, Jakarta, Indonesia. Email: Yvonne.handajani@atmajaya.ac.id; 2 MD, PhD (Neurology), Department of Neurology, School of Medicine and Health Sciences, Atma Jaya Catholic University of Indonesia, Ageing Research Centre, Atma Jaya Catholic University of Indonesia, Jalan Pluit Raya No.2, Jakarta, Indonesia.; 3 MD, MSc (International Primary Care), Center for the Study of Sustainable Community, Atma Jaya Catholic University of Indonesia, Ageing Research Centre, Atma Jaya Catholic University of Indonesia Jalan Pluit Raya No.2, Jakarta, Indonesia.; 4 MD, MS, Ageing Research Centre, Atma Jaya Catholic University of Indonesia Jalan Pluit Raya No.2, Jakarta, Indonesia.; 5 MD, School of Medicine and Health Sciences, Atma Jaya Catholic University of Indonesia, Jalan Pluit Raya No.2, Jakarta, Indonesia.; 6 PhD, Department of Gerontology, University of Southampton University Road, SO17 1BJ Southampton United Kingdom.

**Keywords:** Elderly, Risk factors, Cognitive impairment, Sarcopenia

## Abstract

**Introduction:**

Sarcopenia has many risk factors and an increased likelihood of adverse effects. However, the exact mechanism of how these risk factors cause sarcopenia remains to be elucidated. This study aimed to evaluate the association between physical components, olfactory function impairment and sarcopenia in urban communities.

**Methods:**

This cross-sectional study involved 334 participants aged 60 years and above residing in urban villages in West Jakarta. The medical history, physical ability, cognitive function and olfactory function of participants were examined. Physical ability was assessed based on activities of daily living and instrumental activities of daily living (IADLs). Regarding olfactory function, six scratch test-type odours common in Indonesia were used. Sarcopenia was measured based on muscle mass, muscle strength and physical performance. Binary logistic analysis and backward logistic regression analysis were utilised to explore the association of sarcopenia with the other variables.

**Results:**

Sarcopenia was significantly associated with older age (adjusted odds ratio [AOR]=2.613, 95% confidence interval [CI] = 1.471-4.640), obesity (AOR=0.190 (0.110-0.329), impaired olfactory function (AOR=1.822, 95% CI=1.086-3.056) and frailty (AOR=3.117, 95% CI=1.375-7.064).

**Conclusion:**

The associated factors of sarcopenia include olfactory function, older age, unemployment, impaired IADLs, obesity and frailty.

## Introduction

As ageing is the biological consequence of cellular and molecular injuries over time, it gradually declines physical and mental abilities, subsequently increasing the risk of illnesses and death.^1^ In terms of physical ability, the decline in motor function, including weaker muscles, unsteadiness and easy fatigability, is related to old age, causing progressive disorders such as sarcopenia. Sarcopenia, defined by the occurrence of progressive loss of skeletal muscle mass and characterised by weak and unsteady muscles, may lead to falls, fractures and, potentially, death. In terms of mental ability, ageing deteriorates many cognitive domains, which may worsen physical ability.^2^ This may occur due to engagement in physical activities designed to involve problemsolving, which can help individuals develop procedural knowledge. These activities can foster immediate response strategies and enhance longterm abilities to regulate thoughts and actions.^3^

As people with sarcopenia tend to have a higher likelihood of falls, fractures, physical disabilities and death, many risk factors of sarcopenia have been studied, including pathological mechanisms such as inflammation, metabolic disease and cardiovascular disease.^4^ Basile et al.^4^ postulated a bidirectional association of cognitive and motor impairments and showed that cognitive decline may have a negative impact on balance, strength and walking speed. Similarly, a previous systematic review revealed that mild cognitive impairment substantially affected gait and balance.^4^ However, whether there is any association between cognitive impairment, sarcopenia and ageing remains unclear.^5^ To date, studies on neurocognitive function, such as olfactory function, remain scarce. Although there are uncertainties regarding the exact mechanism, sarcopenia has also been associated with olfactory deficits.^6^ With age, there is a decline in olfactory function and perceived intensity. Olfactory function is also associated with impairments in episodic memory and function.^7^

Many studies have shown an association between sarcopenia subdomains and cognitive impairment. Muscle mass, muscle strength and physical performance are subdomains of the causes of sarcopenia. Through behavioural changes, decreased muscle mass affects the cognitive function of older adults.^8^ Decreased muscle mass leads to reduced function and is associated with pathological mechanisms, including increased inflammatory and oxidative stress and vascular issues associated with cognitive impairment.^9^ Relative to cognitive function, the mechanism involving gait speed is that slow gait speed and lower cognitive function share the same neural pathways. The hippocampus plays a role in walking and cognition due to its involvement in spatial orientation and memory.^10^ These predictors should also be considered from every domain of the sarcopenia index because they have different mechanisms. Understanding these mechanisms can help in understanding sarcopenia more comprehensively. Studies on sarcopenia, especially those that consider predictors from the subdomains of sarcopenia, are necessary to enhance the understanding of this condition.

Our study aimed to evaluate the effects of sarcopenia indices on physical and neurocognitive factors among urban community-dwelling older adults and determine how each domain of sarcopenia is associated with physical components and olfactory function.

## Methods

### Study setting and population

This cross-sectional study involved 334 older adults aged 60 years and above living in one of the urban villages in West Jakarta. Participants were selected via stratified random sampling from each neighbourhood. There are nine neighbourhoods in the selected urban villages. Data were collected from each neighbourhood for a day. Participants included older individuals involved in a part of an active ageing research project, selected according to the following inclusion criteria: (1) age of 60 years and above and (2) ability to come to the data collection site. Individuals with a history of diabetes mellitus were excluded from the study.

We selected the data collection site. Trained interviewers, cadre, urban village officers and participants came to the selected data collection site. Trained interviewers evaluated participants at the data collection site. Informed consent was obtained prior to the interviews. After data collection, data entry and analysis were conducted at the research centre.

### Study variables

Participants’ medical history, general physical examination findings, cognitive function and olfactory function were examined. All variables were analysed as dichotomous.

Physical ability was assessed based on activities of daily living (ADLs) and instrumental activities of daily living (IADLs) and categorised into impaired and normal ADLs and IADLs. The European Working Group on Sarcopenia in Older People (EWGSOP2) interprets sarcopenia using three indices: muscle strength (assessed using handgrip strength), muscle mass (assessed using bioelectrical impedance analysis [BIA]) and physical performance (assessed using gait speed). Handgrip strength was measured using the grip dynamometer model TK-1201 (Takei Kiki Kogyo, Japan).^11^ A handgrip strength under 27 kg for men or 16 kg for women was considered to indicate low muscle strength.^12^ BIA was conducted using the InBody Dial device (Korea).^11^ An appendicular skeletal muscle mass under 20 kg for men or 15 kg for women was deemed as low. A walking speed up to 0.76 m/s for both sexes was deemed as slow.^12^ A 4-m walking speed test was conducted to measure gait speed. Sarcopenia was diagnosed when two indices were met: low muscle strength and mass. Severe sarcopenia was diagnosed when physical performance was poor in addition to sarcopenia. Both were categorised as sarcopenia.^12^

Participants’ ages were grouped into 70 years and over and under 69 years. Educational status was categorised as below 6 years and at least 6 years of education. Trained interviewers inquired about smoking, chronic illnesses (according to the healthcare provider’s assessment) and the frequency and extent of exercise. Exercise referred to engaging in physical activity for at least 30 min per session, repeated a minimum of 3 days per week.^13^ Body mass index (BMI) was measured as body weight divided by the square of height and then classified as obese and not obese according to the World Health Organization BMI classification for Asia Pacific.^14^

The cognitive function test was conducted through the Consortium to Establish a Registry for Alzheimer’s Disease-Neuropsychological Assessment Battery (CERAD-NAB) and the Montreal Cognitive Assessment-Indonesian Version (MoCA-INA). The CERAD-NAB evaluates cognition, including word list memory, recall and recognition; the Boston Naming Test-Short Form (BNT-15) score; verbal fluency; and constructional praxis. The MoCA-INA is composed of eight domains, with scores lower than 16 considered abnormal.

In contrast to previous studies, this study was conducted using six common odours in Indonesia: lemon, orange, eucalyptus oil, menthol, lemongrass and jasmine.^6,15^ We requested a third party to create a scratch test-type odour and label it numerically. Participants were given 10 s to sniff each odour and then instructed to recognise each scent. There were four options available for each odour. A 30-s break was designated between each odour. Impairment was considered when the score was below 4. Conversely, blood pressure measurements were adapted from the VIII Joint National Committee on High Blood Pressure.^16^ Smoking was categorised as current smoker and not a smoker.

### Statistical analysis

A binary logistic analysis was used to compare the characteristics between groups and a backward logistic regression analysis to explore the association of sarcopenia with the independent variables, including age and sex, with the results presented as adjusted odds ratios (AORs). The P-value for the analysed data was considered significant when it was less than 0.05. The data were analysed using IBM SPSS (IBM, New York, USA).

## Results

### Participant characteristics

Among the 334 participants in this study, the mean age was 67.14±5.486 years. Approximately 76% were not working, and the majority had normal functional ability (76.6% had normal ADLs, while 87.4% had normal IADLs). Most of the participants (54.8%) were not obese. Impairment or abnormal test findings were predominantly found, including verbal fluency (79.9%), word list recall (77.5%), word list recognition (97.3%), visuoconstructional praxis (83.8%) and the Boston Naming Test score (91.6%). In contrast, word list memory (64.7%), global cognition based on the MoCA-INA score (56.9%) and olfactory function (51.5%) were normal. Most participants were not frail (85.3%).

Among the 334 participants, 41.6% were sarcopenic, and 25.7% were severely sarcopenic. After analysing each index, we found that 56.6% had low muscle mass; 67.4% had low muscle strength; and 46.7% had poor physical performance.

### Associated factors of sarcopenia

The bivariate analysis showed that sarcopenia was significantly associated with older age, occupation, impaired ADLs, impaired IADLs, obesity, impaired global cognition, impaired olfactory function and frailty (Table 1).

**Table 1:** Characteristics and bivariate analysis of the factors associated with sarcopenia.

Variables	Frequency (%)^#^	Sarcopenia status (%)+		P	Unadjusted OR (95% CI)
		Normal	Sarcopenia		
Age of ≥70 years	99 (29.6)	35 (17.9)	64 (46)	<0.001	3.901 (2.378-6.399)
Unemployment	254	134 (68.7)	120 (86.3)	<0.001	0.348 (0.197-0.616)
Impaired ADLs	78 (23.4)	36 (18.5)	42 (30.2)	<0.05	1.912 (1.146-3.190)
Impaired IADLs	42 (12.6)	12 (6.2)	30 (21.6)	<0.001	4.197 (2.063-8.539)
Obesity	151 (45.2)	119 (61)	32 (24.6)	<0.001	0.191 (0.117-0.311)
Impaired verbal fluency	267 (79.9)	157 (80.5)	110 (79.5)	>0.05	0.918 (0.534-1.577)
Impaired BNT performance	306 (91.6)	177 (90.8)	129 (92.8)	>0.05	1.312 (0.586-2.936)
Impaired global cognition	144 (43.1)	73 (37.4)	71 (51.1)	<0.05	1.745 (1.122-2.713)
Impaired word list memory	118 (35.3)	63 (32.3)	55 (39.6)	>0.05	1.372 (0.872-2.159)
Impaired word list recall	259 (77.5)	146 (74.9)	113 (81.3)	>0.05	1.459 (0.854-2.491)
Impaired word list recognition	325 (97.3)	188 (96.4)	137 (98.6)	>0.05	2.551 (0.522-12.467)
Impaired visuoconstruction	280 (83.8)	162 (83.1)	118 (84.9)	>0.05	1.145 (0.630-2.078)
Impaired olfactory function	162 (48.5)	76 (39)	86 (61.9)	<0.001	2.541 (1.625-3.973)
Frailty	49 (14.7)	14 (7.2)	35 (25.2)	<0.001	4.351 (2.237-8.461)

# Proportion of the cohort (among all population), +proportion among each subgroup (normal and sarcopenia) OR: odds ratio, CI: confidence interval, ADLs: activities of daily living, IADLs: instrumental activities of daily living, BNT: Boston Naming Test

Sarcopenia was significantly associated with older age (AOR=2.613, 95% confidence interval [CI] = 1.471-4.640), frailty (AOR=3.117, 95% CI=1.375-7.064), impaired olfactory function (AOR=1.822, 95% CI=1.086-3.056) and obesity (AOR=0.190, 95% CI=0.110-0.329) (Table 2).

**Table 2:** Backward logistic regression analysis of the factors associated with sarcopenia.

Variables	AOR (95% CI)	P
Age of ≥70 years	2.613 (1.471-4.640)	0.001
Frailty	3.117 (1.375-7.064)	<0.05
Impaired olfactory function	1.822 (1.086-3.056)	<0.05
Obesity	0.190 (0.110-0.329)	<0.05

Reference: Age under 70 years, no frailty, normal olfactory function and normal body mass index (BMI) CI: confidence interval, AOR: adjusted odds ratio

Regarding the sarcopenia indices, low muscle mass was strongly associated with older age (AOR=2.384, 95% CI= 1.267-4.486), impaired IADLs (AOR=3.727, 95% CI= 1.548-8.970) and obesity (AOR=0.039, 95% CI=0.011-0.131) (Table 3).

**Table 3:** Backward logistic regression analysis of the factors associated with muscle mass.

Variables	AOR (95% CI)	P
Age of ≥70 years	2.384 (1.267-4.486)	<0.05
Impaired IADLs	3.727 (1.548-8.970)	<0.005
Obesity	0.039 (0.011-0.131)	<0.001

Reference: Age under 70 years, normal IADLs and normal body mass index (BMI) CI: confidence interval, AOR: adjusted odds ratio, IADLs: instrumental activities of daily living

Low muscle strength was strongly associated with older age (AOR=2.050, 95% CI= 1.101–3.819), frailty (AOR=17.291, 95% CI=2.283-130.976) and unemployment (AOR=1.838, 95% CI=1.061-3.181) (Table 4).

**Table 4:** Backward logistic regression analysis of the factors associated with muscle strength.

Variables	AOR (95% CI)	P
Age of ≥70 years	2.050 (1.101-3.819)	<0.05
Frailty	17.291 (2.283-130.976)	<0.05
Unemployment	0.544 (0.314-0.942)	<0.05

Reference: Age under 70 years, no frailty and employment CI: confidence interval, AOR: adjusted odds ratio

Poor physical performance was significantly associated with impaired ADLs (AOR=7.440, 95% CI=3.772-14.676) and impaired IADLs (AOR=7.189, 95% CI=3.088-16.734) (Table 5).

**Table 5:** Backward logistic regression analysis of the factors associated with physical performance.

Variables	AOR (95% CI)	P
Impaired ADLs	7.440 (3.772-14.676)	<0.001
Impaired IADLs	7.189 (3.088-16.734)	<0.001

Reference: Normal ADLs and normal IADLs CI: confidence interval, AOR: adjusted odds ratio, ADLs: activities of daily living, IADLs: instrumental activities of daily living

## Discussion

This section will discuss the physical components and olfactory functions associated with sarcopenia and its indices. In our study, 41% of the participants had sarcopenia. A previous meta-analysis showed that the prevalence of sarcopenia was 10% and 18% according to the EWGSOP2 and Asian Working Group for Sarcopenia criteria, respectively, among a population of 692,056 individuals with a mean age of 68.5 years.^17^ Likewise, Khongsri et al. found that the prevalence of sarcopenia in older adults ranged from 22% to 30%. Nevertheless, as the definition and measurement of sarcopenia vary, it is challenging to evaluate the prevalence homogeneously.^18^

The present study showed several significant associations between older age, unemployment, impaired IADLs, obesity, impaired olfactory function, frailty and sarcopenia. The individuals aged 70 years and above had a notable risk of sarcopenia (P=0.001, AOR=2.613). Aged muscle exhibits changes in muscle cell types, mitochondrial function, nicotinamide adenine dinucleotide metabolism, myokines and gut microbiota compared to young or healthy aged muscle.^19^ This result is consistent with several reports demonstrating that older age is significantly associated with a higher risk of primary sarcopenia.^20,21^

In this study, the employed participants included those receiving payment for their work, whereas the unemployed participants included those not receiving payment for their work, such as housewives, volunteers and social workers. Unemployment was significantly associated with a lower risk of sarcopenia, which is inconsistent with the findings of Dorosty et al., wherein the unemployment group showed a higher risk of sarcopenia. Further exploration revealed that older adults whose occupation involved insufficient physical activity were at risk of sarcopenia.^22^ As a result, physical activity is considered a quite important factor, even during occupational work. In our study, doing housework was not considered an occupation. This emphasises the concept of housework as a light-to-moderate-intensity physical activity and a healthy lifestyle.

Our study also showed that obesity was a protective factor against sarcopenia and low muscle mass, which is in accordance with the findings by Zhang et al. revealing an inverse association between sarcopenia and muscle mass.^23^ Among obese individuals, the frequently observed high protein consumption and elevated oestrogen levels may protect them from muscle loss and become protective factors for sarcopenia. Regarding oestrogen, the anabolic inhibition of muscle via conversion of androgen to oestrogen might protect lean mass.^24^ Nevertheless, the inverse association between obesity and sarcopenia might be distorted by muscle mass. After adjustment, a high BMI is associated with the risk of sarcopenia, suggesting that a large amount of fat is not a protective factor against sarcopenia.^7^

This study explored the neurocognitive factors associated with sarcopenia, such as olfactory function, which can be easily detected earlier. Handajani et al. and Harita et al. demonstrated similar findings, where olfactory disorders were associated with sarcopenia.^8,25^ Olfactory disorders measured using the Open Essence Test are associated with the sarcopenia indices even after adjustments.^14^ As the ability to enjoy flavours declines, so does appetite. This, along with other forms of interest in food, raises the risk of reduced activity levels, and a lower body protein index will lead to a lower index of sarcopenia, such as muscle mass and muscle grip.^25^ However, research explaining the precise mechanism by which olfactory function affects sarcopenia is still insufficient.

Frailty and sarcopenia, as the two key geriatric syndromes in the ageing process, share a similar proposed pathophysiology.^25^ Changes in innate immunity during ageing can contribute to the development of frailty and sarcopenia in the future. This is mainly due to the inflammation during ageing, called inflammaging, which refers to an increased amount of low-grade inflammation attributed to ageing. It is characterised by escalating levels of pro-inflammatory cytokines and reduced levels of anti-inflammatory cytokines, leading to degradation of muscle and inhibition of muscle synthesis.^26^

The mechanism underlying the mutual association between ADL disability and muscle strength is complex.^27^ ADL impairment increases the probability of having low muscle strength, mediated by higher levels of inflammation. Functional activities such as eating and shopping might be challenging for impaired individuals, contributing to deficient nutrients and declining muscle strength. Moreover, these individuals tend to have other illnesses, such as long-term stress, mental disorders and cognitive impairment, which may contribute to reduced muscle strength.^28^

In community-dwelling older adults, slower gait speed is strongly linked to lower basic and advanced daily functions compared with other indices. Strength, balance, dexterity and cognitive control are evaluated in the Short Physical Performance Battery (SPPB), similar to ADLs and IADLs.^29^ Low SPPB scores reveal skeletal muscle function problems, which can be worsened by structural and neurological changes, contributing to low functional abilities.^27^

It is imperative to gain a deeper understanding of each sarcopenia index to prevent a higher risk of sarcopenia.^6^ This study revealed several significant associations with each sarcopenia domain. First, low muscle mass was strongly associated with older age, impaired IADLs and obesity (Table 3). Second, low muscle strength was strongly associated with older age, unemployment and frailty (Table 4). Lastly, poor physical performance was significantly associated with impaired ADLs and impaired IADLs (Table 5). In their study, Sui et al. revealed that worse gait speed and timed chair stand test findings were found in the cognitively impaired category.^30^ Other studies have also shown that poor cognitive function is associated with a more significant gait speed decline over time.^31^

This study has limitations. First, due to the cross-sectional study design, the participants were not followed up. Additionally, this study included only older people who were able to visit the data collection site. Second, due to a lack of tools, this study measured only one type of physical performance: the timed chair stand test score. Third, we used the EWGS-recommended cutoff values for each sarcopenia index. These values were made for Europeans and may not apply to all Asians, as muscle index differs by ethnicity.

## Conclusion

There are significant associations between sarcopenia and older age, obesity, impaired olfactory function and frailty. Sarcopenia is characterised by three indices: muscle mass, muscle strength and physical performance. Low muscle mass is significantly associated with older age, impaired IADLs and obesity. Low muscle strength is significantly associated with older age, unemployment and frailty. Poor physical performance is significantly associated with impaired ADLs and impaired IADLs.

Based on the findings, it can be concluded that sarcopenia is influenced by neurocognitive factors such as olfactory function, as well as physical factors such as older age, obesity and frailty. Furthermore, the following factors affecting physical performance also affect the three indices of sarcopenia: old age, IADL impairment, obesity, unemployment, daily activity component (ADLs and IADLs) and frailty. However, further research is needed to examine how olfactory function is specifically associated with sarcopenia.

## Data sharing statement

The supporting data are available from the corresponding author upon reasonable request.

**How does this paper make a difference in general practice?**

- This paper would help general practitioner to plan the right cost-effective treatment of elderly patient with geriatric syndrome.
- This paper can help general practitioner to enhance a person-centred care to elderly group.
- This paper highlights the importance of nutrition in elderly.
- This paper may give insight to generate the guideline for sarcopenia in primary care setting.
- This paper would help future researcher to find the right approach to prevent sarcopenia in elderly group

## References

[1] World Health Organization. (2022). Ageing and Health..

[2] Murman D (2015). The impact of age on cognition.. Semin Hear..

[3] Latino F, Tafuri F (2024). Physical activity and cognitive functioning.. Medicina..

[4] Basile G, Sardella A (2021). From cognitive to motor impairment and from sarcopenia to cognitive impairment: a bidirectional pathway towards frailty and disability.. Aging Clin Exp Res..

[5] Yuan S, Larsson SC (2023). Epidemiology of sarcopenia: prevalence, risk factors, and consequences.. Metabolism..

[6] Handajani Y, Butterfill E, Hengky A, Sugiyono S, Lamadong V, Turana Y (2023). Sarcopenia and impairment in global cognitive, delayed memory, and olfactory function, among community-dwelling adults, in Jakarta, Indonesia: active aging study.. Tzu Chi Med J..

[7] Delgado-Lima AH, Bouhaben J, Martfnez-Zujeros S, Pallardo-Rodil B, Gomez-Pavon J, Delgado-Losada ML (2023). Could olfactory identification be a prognostic factor in detecting cognitive impairment risk in the elderly?. GeroScience..

[8] Burns JM, Johnson DK, Watts A, Swerdlow RH, Brooks WM (2010). Reduced lean mass in early Alzheimer disease and its association with brain atrophy.. Arch Neurol..

[9] Cavalcante BR, Falck RS, Liu-Ambrose T (2023). “May the force (and size) be with you”: muscle mass and function are important risk factors for cognitive decline and dementia.. J Nutr Health Aging..

[10] Rosano C, Snitz BE (2018). Predicting dementia from decline in gait speed: are we there yet?. J Am Geriatr Soc..

[11] Sri-on J, Fusakul Y, Kredarunsooksree T, Paksopis T, Ruangsiri R (2022). The prevalence and risk factors of sarcopenia among Thai community-dwelling older adults as defined by the Asian Working Group for Sarcopenia (AWGS-2019) criteria: a cross-sectional study.. BMC Geriatr..

[12] Cruz-Jentoft AJ, Bahat G, Bauer J (2019). Sarcopenia: revised European consensus on definition and diagnosis.. Age Ageing..

[13] Kementerian Kesehatan RI (2017). Buku Saku Ayo Bergerak, Lawan Obesitas!.

[14] World Health Organization. (2000). The Asia-Pacific perspective: redefining obesity and its treatment..

[15] Luhur JJ, Handajani YS, Handajani TY (2012). Determination of familiar odours for standard examination of olfactory function of the elderly in Jakarta.. Neurona..

[16] James PA, Oparil S, Carter BL (2014). 2014 Evidence-based guideline for the management of high blood pressure in adults Report From the Panel Members Appointed to the Eighth Joint National Committee (JNC 8).. JAMA..

[17] Petermann-Rocha F, Balntzi V, Gray SR (2022). Global prevalence of sarcopenia and severe sarcopenia: a systematic review and metaanalysis.. J Cachexia Sarcopenia Muscle..

[18] Khongsri N, Tongsuntud S, Limampai P, Kuptniratsaikul V (2016). The prevalence of sarcopenia and related factors in a community-dwelling elders Thai population.. Osteoporos Sarcopenia..

[19] Cai L, Shi L, Peng Z, Sun Y, Chen J (2023). Ageing of skeletal muscle extracellular matrix and mitochondria: finding a potential link.. Ann Med..

[20] Gao Q, Hu K, Yan C (2021). Associated factors of sarcopenia in community-dwelling older adults: a systematic review and meta-analysis.. Nutrients..

[21] Therakomen V, Petchlorlian A, Lakananurak N (2020). Prevalence and risk factors of primary sarcopenia in community-dwelling outpatient elderly: a cross-sectional study.. Sci Rep..

[22] Dorosty A, Arero G, Chamar M, Tavakoli S (2016). Prevalence of sarcopenia and its association with socioeconomic status among the elderly in Tehran.. Ethiop J Health Sci..

[23] Zhang Y, Chen X, Hou L (2020). Prevalence and risk factors governing the loss of muscle function in elderly sarcopenia patients: a longitudinal study in China with 4 years of follow-up.. J Nutr Health Aging..

[24] Cheng Q, Zhu X, Zhang X (2014). A crosssectional study of loss of muscle mass corresponding to sarcopenia in healthy Chinese men and women: reference values, prevalence, and association with bone mass.. J Bone Miner Metab..

[25] Harita M, Miwa T, Shiga H (2019). Association of olfactory impairment with indexes of sarcopenia and frailty in community-dwelling older adults.. Geriatr Gerontol Int..

[26] Shimada H, Tsutsumimoto K, Doi T (2021). Effect of sarcopenia status on disability incidence among Japanese older adults.. J Am Med Dir Assoc..

[27] Corsonello A, Lattanzio F, Pedone C (2012). Prognostic significance of the short physical performance battery in older patients discharged from acute care hospitals.. Rejuvenation Res..

[28] Khow KSF, Yu SCY (2017). An overview of frailty and sarcopenia in older people.. J Aging Geriatr Med..

[29] Wang DXM, Yao J, Zirek Y, Reijnierse EM, Maier AB (2020). Muscle mass, strength, and physical performance predicting activities of daily living: a meta-analysis.. J Cachexia Sarcopenia Muscle..

[30] Sui SX, Williams LJ, Holloway-Kew KL, Hyde NK, Pasco JA (2020). Skeletal muscle health and cognitive function: a narrative review.. Int J MolSci..

[31] Watson NL, Rosano C, Boudreau RM (2010). Executive function, memory, and gait speed decline in well-functioning older adults.. J Gerontol A Biol Sci Med Sci..

